# Primary cilia and inflammatory response: unveiling new mechanisms in osteoarthritis progression

**DOI:** 10.3389/ebm.2025.10490

**Published:** 2025-04-28

**Authors:** Yuyan Sun, Ziyu Luo, Yuanyuan Fu, ThaiNamanh Ngo, Wen Wang, Yuanrong Wang, Ying Kong

**Affiliations:** Department of Rehabilitation, The Second Xiangya Hospital, Central South University, Changsha, China

**Keywords:** osteoarthritis, primary cilia, inflammatory responses, chondrocytes, inflammatory signaling pathways

## Abstract

Osteoarthritis (OA) is a common degenerative joint disease that can lead to chronic pain and disability. The pathogenesis of OA involves chronic low-grade inflammation, characterized by the degradation of chondrocytes, inflammation of the synovium, and systemic low-grade inflammation. This inflammatory response accelerates the progression of OA and contributes to pain and functional impairment. Primary cilia play a crucial role in cellular signal transduction and the maintenance of cartilage matrix homeostasis, and their dysfunction is closely linked to inflammatory responses. Given these roles, primary cilia may significantly contribute to the pathogenesis of OA. This review explores inflammation-associated signaling pathways in OA, including NF-κB, MAPK, JAK/STAT, and PI3K/AKT/mTOR signaling. In addition, we place particular emphasis on cilia-mediated inflammatory modulation in OA. Primary cilia mediate chondrocyte responses to mechanical loading and inflammatory cytokines via pathways including NF-κB, MAPK, TRPV4, and Hedgehog signaling. Notably, alterations in the length and incidence of primary cilia in chondrocytes during OA further underscore their potential role in disease pathogenesis. The identification of biomarkers and therapeutic targets related to primary cilia and inflammatory pathways offers new potential for the treatment and management of OA.

## Impact statement

This review is crucial for the field of osteoarthritis research as it unveils the critical role of primary cilia in the inflammatory pathways of osteoarthritis, a previously under explored area. By investigating the complex molecular mechanisms linking primary cilia and inflammation signaling, the review provides new insights into how primary cilia contribute to chronic inflammation and cartilage degradation. These findings may prove valuable for the early diagnosis and targeted treatment of osteoarthritis.

## Introduction

Osteoarthritis (OA) is a prevalent chronic joint disease characterized by functional impairment, pain, cartilage degeneration, synovial inflammation, and structural alterations in the subchondral bone, including sclerosis, osteophyte formation, and cystic lesions [1]. As a leading cause of disability and chronic pain globally, OA affects over 500 million people (7% of the world’s population), with a particularly high incidence in the elderly and women [2, 3]. This condition significantly diminishes mobility, physical function, and quality of life in older adults [4]. Despite its widespread impact, the treatment of OA remains challenging, primarily due to the limited understanding of its pathogenesis and the mechanisms underlying its progression [5]. Therefore, there is an urgent need for in-depth research into the key signaling pathways and molecular mechanisms involved in OA, along with the identification of potential biomarkers and therapeutic targets for its various stages.

OA was traditionally regarded as a non-inflammatory joint disease, but recent studies have highlighted the critical role of inflammatory responses in its pathogenesis [6]. Unlike the acute inflammation observed in autoimmune diseases like rheumatoid arthritis (RA), OA is characterized by low-grade, chronic inflammation [4]. Table 1 presents a comparison of osteoarthritis with other types of joint inflammations. A hallmark of the pathological process of OA is synovitis, which involves synovial hyperplasia and the infiltration of inflammatory cells into the synovium. These immune cells release pro-inflammatory factors such as interleukin-1β (IL-1β), tumor necrosis factor-α (TNF-α), and nitric oxide (NO). This inflammatory factors subsequently activate matrix metalloproteinases (MMPs) and aggrecanases, further accelerating cartilage degradation. Ultimately, these processes lead to joint pain and functional impairment [6, 15]. Synovial fluid serves as a multi-functional mediator between the synovium and articular cartilage, and the inflammatory microenvironment of the synovial fluid plays a pivotal role in regulating OA pathogenesis [16]. OA synovial fluid not only drives macrophage polarization toward the pro-inflammatory M1 phenotype [17] but also upregulate catabolic enzymes matrix metalloproteinases (MMPs) [16]. Additionally, mast cells—one of the dominant infiltrating immune cell populations in OA synovium—contribute to synovitis progression and osteophyte formation through the release of pro-inflammatory mediators [18]. In addition to local joint inflammation, systemic inflammation also plays a significant role in the pathogenesis of OA [6]. For instance, obesity, a known risk factor for OA, not only increases the mechanical load on the joints but also triggers systemic low-grade inflammation. This inflammation is mediated by inflammatory factors released by adipose tissue, such as adipokines and other pro-inflammatory cytokines, which further promote the onset of OA [6, 15, 19, 20]. Therefore, inflammation accelerates the progression of OA by fostering chondrocyte degradation, synovial inflammation, and systemic low-grade inflammation, thereby causing pain and functional impairment in patients.

**TABLE 1:** Comparison of different types of joint inflammations.

Disease	Osteoarthritis (OA)	Rheumatoid arthritis (RA)	Psoriatic arthritis (PsA)	Gouty arthritis (GA)	Ankylosing spondylitis (AS)
Nature of Disease	Degenerative joint disease with chronic low-grade inflammation	Chronic inflammatory autoimmune disease	Chronic inflammatory autoimmune disease	Autoimmune and metabolic disease with acute inflammation	Chronic inflammatory autoimmune disease, spondyloarthritis
Mechanism of Inflammation	Joint damage or overuse triggers an immune response, leading to local tissue damage, failed tissue repair, and low-grade inflammation within the joint	Persistent autoimmune response causes synovial inflammation, with elevated pro-inflammatory cytokines (e.g., IL-6, TNF-α) and autoantibodies [e.g., Rheumatoid factor (RF), Anti-citrullinated protein antibodies (ACPA)]	Interaction of genetic susceptibility and environmental triggers leads to dysregulation of immune-inflammatory pathways	Elevated serum uric acid levels lead to deposition of monosodium urate crystals in joint tissues, activating an inflammatory response	Genetic factors (e.g., HLA-B27) and autoimmune response, involving Th17-related inflammatory signaling
Inflammatory Cell Infiltration	Macrophages, mast cells	T cells, B cells, macrophages, fibroblasts	Macrophages, T cells, neutrophils, dendritic cells	Lymphocytes, dendritic cells, macrophages, neutrophils, mast cells	Macrophages, neutrophils, mast cells
Inflammatory Markers	IL-1β, TNF-α, IL-6, MMPs, C-reactive protein (CRP)	CRP, Erythrocyte sedimentation rate (ESR), RF, ACPA, IL-6, TNF-α, IFN-γ, Th17A	IL-17, IL-23	IL-1β, TNF-α, IL-6	IL-23, IL-17
Affected Sites	Weight-bearing joints (knees, hips, spine, distal interphalangeal joints of the hand)	Hands, arms, knees	Asymmetric involvement of large joints, monoarticular or oligoarticular, enthesitis, and dactylitis	Monoarticular or polyarticular, commonly affecting the big toe	Sacroiliac joints, spine, hip joints, knee joints
Disease Progression	Cartilage degeneration, osteophyte formation, joint capsule thickening, synovial inflammation	Chronic synovial joint inflammation, pannus formation, bone erosion, and joint destruction	Skin manifestations often precede joint symptoms	Acute attacks with symptom-free intervals	Chronic back pain and stiffness, potentially progressing to spinal ankylosis
Treatment Strategies	Nonsteroidal anti-inflammatory drugs (NSAIDs), physical therapy, analgesics, steroids, hyaluronic acid	Disease-modifying antirheumatic drugs (DMARDs), NSAIDs, immunosuppressive corticosteroids, physical therapy	NSAIDs, biologics, anti-TNF-α agents, anti-IL-17 agents, anti-IL-12/IL-23, skin treatments	Colchicine, NSAIDs, corticosteroids, IL-1β inhibitors, urate-lowering drugs	Physical therapy, NSAIDs, TNF inhibitors, IL-17 inhibitors, JAK inhibitors
References	Robinson WH et al. (2016) [6]	Lin YJ et al. (2020) [7] Scott DL et al. (2010) [8]	Porta S et al. (2021) [9] Talotta R et al. (2019) [10]	Liu et al. (2023) [11] Cabău G et al. (2020) [12]	Wei et al. (2025) [13] Voruganti A et al. (2020) [14]

Primary cilia are microtubule-based structures that extend from the surface of cells. They are widely distributed across various cell types, including chondrocytes, synovial cells, and other joint tissue cells, and play an important role in sensing and transmitting mechanical and chemical signals in these cells [21]. Growing evidence indicates that primary cilia are essential for chondrocyte signal transduction and the maintenance of cartilage matrix homeostasis, particularly in the pathogenesis of OA [21]. The direction, length, and number of cilia in chondrocytes are closely linked to OA progression, and their dysfunction may contribute to inflammatory responses and cartilage degeneration [22, 23]. Consequently, regulating primary cilia and their associated signaling pathways not only enhances our understanding of OA pathogenesis but also offers potential therapeutic targets for treatment.

In recent years, there has been increasing attention on the role of primary cilia in the inflammatory signaling processes of OA. This review, therefore, focuses on the involvement of primary cilia in OA-related inflammation. It examines the inflammatory signaling pathways in which primary cilia are involved, as well as the bidirectional regulatory mechanisms between inflammation and primary cilia. The goal of this review is to elucidate the potential roles and mechanisms of primary cilia in OA inflammation. We hope that this article will offer new insights for the treatment of OA.

## Inflammatory signaling pathways in osteoarthritis

**FIGURE 1:**
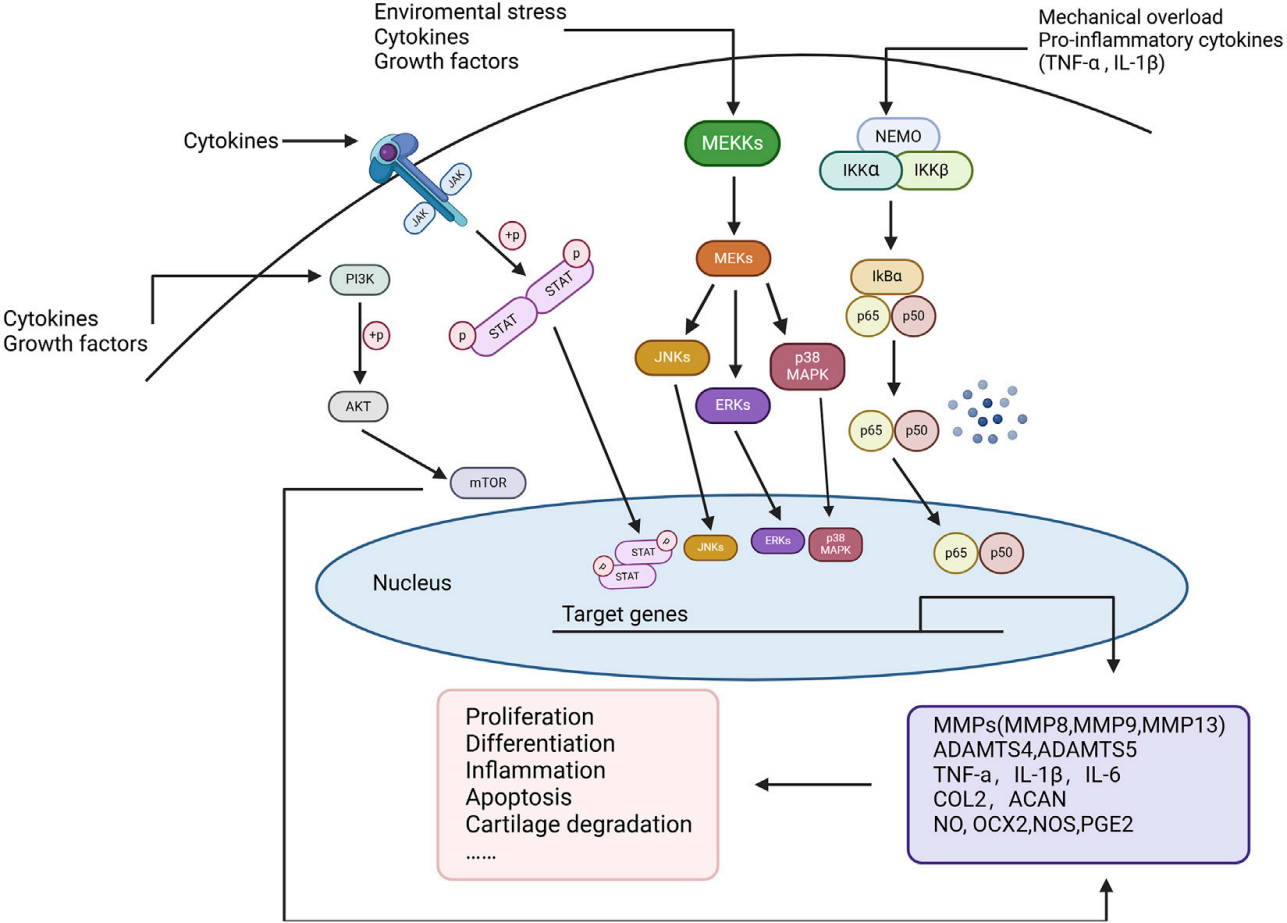
Key inflammatory signaling pathways in OA. Environmental stress, mechanical overload, and pro-inflammatory cytokines (such as TNF-α and IL-1β) activate multiple signaling pathways, including NF-κB, MAPK, JAK/STAT, and PI3K/AKT/mTOR, to regulate cellular inflammatory responses. These pathways, through multi-level phosphorylation cascades, ultimately translocate into the nucleus to regulate the expression of key genes associated with OA progression, such as matrix metalloproteinases (MMPs), ADAMTS enzymes, TNF-α, IL-1β, and IL-6. This process induces cellular proliferation, differentiation, inflammation, apoptosis, and cartilage degradation, playing a pivotal role in OA pathogenesis.

In OA, sustained chronic inflammation is associated with the degradation of articular cartilage and synovial hyperplasia, promoting joint degeneration and triggering pain responses [24]. These inflammatory reactions are mediated by a series of cytokines, chemokines, and enzymes [25]. Below are several key inflammatory signaling pathways closely associated with OA. The major signaling pathways that regulate inflammation in OA cartilage include NF-κB, JAK/STAT, MAPK, and PI3K/AKT/mTOR, among others [25, 26] (Figure 1).

### NF-κB signaling pathway

NF-κB is a family of transcription factors that likely plays a critical role in various biological processes, including immune responses, inflammation, cell differentiation, as well as cell proliferation and apoptosis [27]. NF-κB activation occurs mainly through two pathways: the classical and non-classical pathways. In the classical pathway, various immune mediators, such as pro-inflammatory cytokines (e.g., TNF-α and IL-1β), activate the IKK complex (IKKα/β/γ), leading to the phosphorylation and degradation of IκB proteins. This process releases NF-κB dimers (e.g., p65/p50/RelB), which translocate to the nucleus and initiate the transcription of target genes. This process is rapid and reversible [28, 29]. In contrast, the non-classical pathway primarily involves IKKα activation, which leads to the production of p52 and RelB, activating target gene transcription. This pathway is slower and more sustained [30].

In the pathogenesis of OA, NF-κB is a transcription factor extensively involved in the joint inflammation and tissue-destruction processes [5]. Mechanical receptors on the cell membrane of chondrocytes, cytokine receptors, TNFR, and TLRs, activated by pro-inflammatory mediators, mechanical stress, or fibronectin fragments, induce NF-κB signaling [31]. NF-κB signaling promotes the secretion of various degradative enzymes, such as MMPs (including MMP1, MMP9, MMP13) and a disintegrin and metalloproteinases with thrombospondin motifs (ADAMTS, e.g., ADAMTS5), which lead to cartilage degradation [29]. Moreover, NF-κB mediates the production of catabolic cytokines and chemokines, including TNF-α, IL-1β, IL-6, receptor activator of NF-κB ligand (RANKL), and IL-8. This increases the production of MMPs, reduces the synthesis of collagen and proteoglycans, and enhances NF-κB activation through a positive feedback loop [32]. Additionally, NF-κB promotes joint damage by inducing the synthesis of pro-inflammatory molecules such as nitric oxide (NO), cyclooxygenase-2 (COX-2), nitric oxide synthase (NOS), and prostaglandin E2 (PGE2), thereby contributing to cartilage inflammation, the production of catabolic factors, and chondrocyte apoptosis [33, 34]. Furthermore, the NF-κB pathway interacts with other signaling pathways, such as Wnt, to collectively drive the inflammatory response and tissue degeneration in OA [35].

### MAPK signaling pathway

Mitogen-activated protein kinases (MAPKs) are a group of serine/threonine protein kinases found in eukaryotes [36]. MAPK signaling is activated by extracellular stimuli such as pro-inflammatory cytokines, growth factors, and oxidative stress. This signaling transduces the extracellular signal into the nucleus via a phosphorylation cascade, regulating various cellular processes [37], such as inflammation responses, catabolism, and degradation of the extracellular matrix, that can have a crucial role in OA development [38]. The extracellular signal-regulated kinase (ERK), c-Jun N-terminal kinase (JNK), and p38 MAPK are the most studied MAPK family members involved in OA pathogenesis. Each cascade consists of at least three enzymes to activate the signaling pathway (MAPKK, MAPK kinase, and MAPK) [39]. Under inflammatory conditions, phosphorylation levels of ERK1, JNK, and p38 MAPK are upregulated. This upregulation stimulates downstream transcription factors, thereby promoting the production of inflammatory cytokines and the progression of inflammatory responses [38]. Inflammatory factors like IL-1 can activate the MAPK signaling pathways, subsequently promoting the expression and activity of MMPs and leading to the degradation of the cartilage [40]. Recent studies have confirmed that both JNK and p38 MAPK signaling pathways are associated with chondrocyte apoptosis in OA [41], while the p38 MAPK and ERK1/2 pathways are closely linked to the cross-talk between bone and cartilage [42, 43].

### JAK/STAT signaling pathway

The JAK/STAT signaling pathway consists of three components: Janus kinases (JAKs), signal transducer and activator of transcription proteins (STATs), and tyrosine kinase-associated receptors [44]. This pathway regulates cell proliferation, differentiation, apoptosis, and inflammation [39, 44]. In the pathogenesis of OA, pro-inflammatory cytokines such as IL-1β, IL-6, and TNF-α bind to cell membrane receptors, activating JAK kinases, which in turn phosphorylate STAT transcription factors [45]. The phosphorylated STATs dimerize and translocate to the nucleus, where they bind to promoter regions of target genes to regulate gene expression [44], promoting synovial and cartilage inflammation, as well as the degeneration of cartilage [45]. For example, IL-1β and IL-6 activate JAK/STAT3 signaling in chondrocytes, inducing the expression of MMPs like MMP-13, directly contributing to cartilage matrix degradation and exacerbating cartilage damage [46, 47]. Simultaneously, the JAK/STAT pathway can induce the production of pro-inflammatory factors, such as IL-6, thereby creating a vicious cycle that intensifies synovial inflammation and promotes OA progression [48]. The JAK/STAT signaling pathway also regulates factors such as ELP2 [49] and RANKL [50], which are involved in the differentiation of osteoblasts and osteoclasts. This pathway also induces pathological angiogenesis in subchondral bone through HIF-α/VEGF pathways [51] and factors like EGFL7 [52], thereby exacerbating joint pathological changes.

### PI3K/AKT/mTOR signaling pathway

The PI3K/AKT/mTOR signaling pathway consists of PI3K, AKT, and mTOR. When insulin, glucose, and various growth factors and cytokines stimulate the cell membrane, PI3K is activated. PI3K catalyzes the phosphorylation of phosphatidylinositol-4,5-bisphosphate (PIP2) to produce phosphatidylinositol-3,4,5-trisphosphate (PIP3), which activates the downstream AKT protein [53]. The downstream effector of PI3K, mTOR, also functions as a regulator of pro-inflammatory responses in the synovium [53]. In OA pathology, chondrocytes and synovial cells excessively produce inflammatory mediators such as IL-1β and NO [32]. Stimulation by cytokines like IL-1β rapidly phosphorylates PI3K and AKT, leading to the abnormal activation of the PI3K/AKT pathway [54]. This activation increases the production of MMPs by chondrocytes through multiple downstream target proteins [55]. Additionally, protein kinase A (PKA)/AKT can activate NF-κB by affecting the upstream IκB kinase, leading to the phosphorylation and nuclear translocation of NF-κB p65. The interaction between PI3K/AKT and NF-κB enhances the release of inflammatory factor [53].

## Primary cilium-mediated inflammatory signaling regulation

**FIGURE 2:**
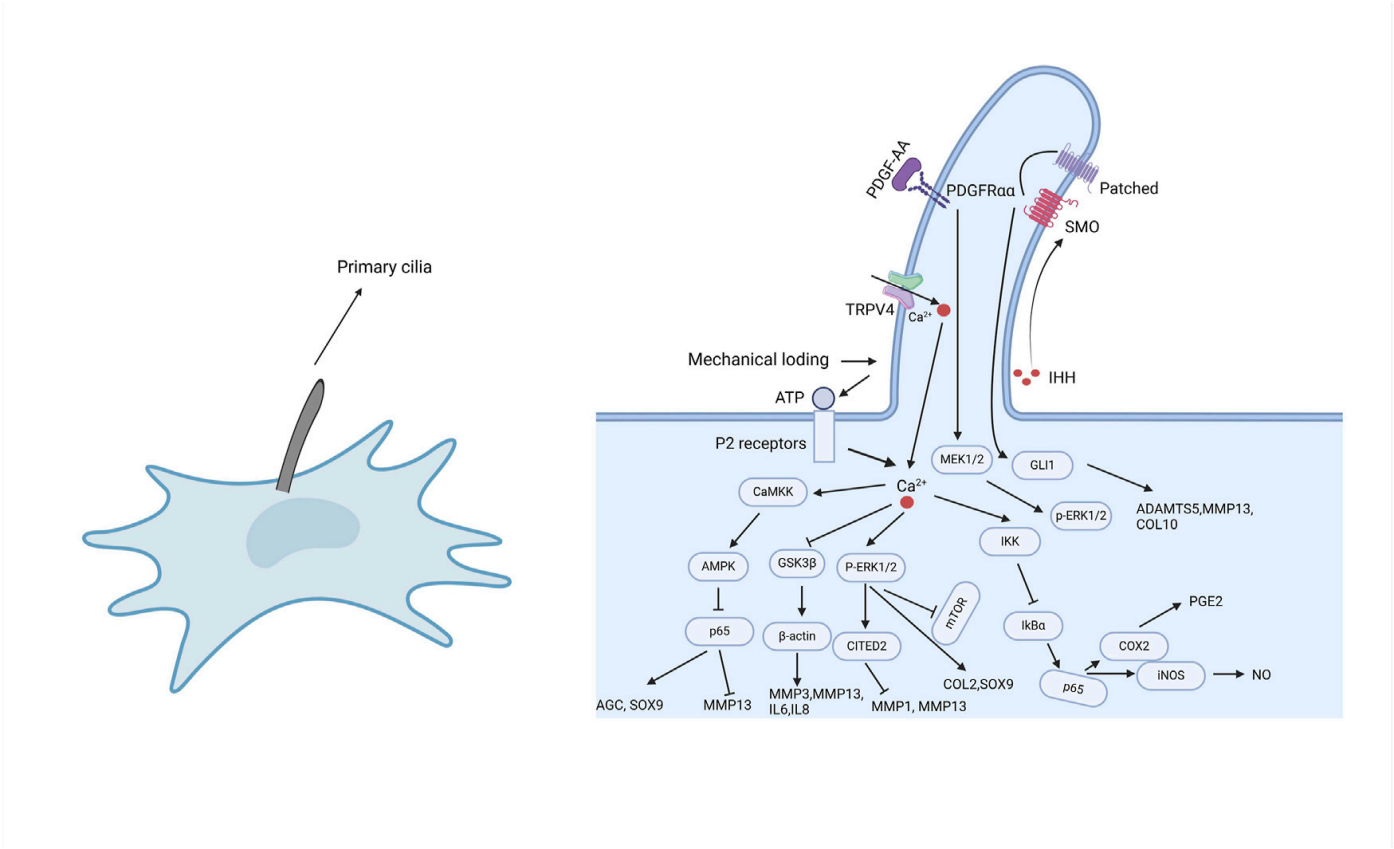
The role of primary cilia in inflammatory responses. As cellular sensory organelles, primary cilia regulate multiple signaling pathways, including NF-κB, MAPK, TRPV4, and Hedgehog pathways. Mechanical loading or external stimuli trigger Ca^2+^ influx through TRPV4 channels, activating a cascade of signaling events, such as the CaMKK-AMPK-NF-κB axis to regulate inflammation, the GSK3β pathway to influence cytoskeletal dynamics, and the P-ERK1/2 pathway to suppress the overexpression of inflammatory factors. Additionally, primary cilia modulate IHH and PDGFRα signaling, contributing to the regulation of MMPs, ADAMTS enzymes, and inflammation-related factors. These signaling pathways work in concert to regulate cartilage homeostasis, inflammatory responses, and the pathological progression of osteoarthritis.

Primary cilia participate in a variety of cellular signaling pathways, with the most commonly involved being Hedgehog, Wnt, platelet-derived growth factor (PDGF), transforming growth factor-β (TGF-β), and mechanotransduction [23, 56]. Abnormalities or dysfunction of primary cilia negatively impact cartilage homeostasis, promoting abnormal cartilage remodeling and early-onset OA [57]. Recent studies have shown that primary cilia play a role in the inflammatory processes of OA [23], interacting with several inflammatory signaling pathways, including NF-κB, TRPV4 (Transient Receptor Potential Vanilloid 4), MAPK, and Hedgehog pathways (Figure 2). These inflammatory pathways are involved in the expression of inflammatory factors and the initiation of inflammatory responses, critically affecting the onset and progression of OA. Below, we will describe in detail the relationships between these signaling pathways and primary cilia (Table 2).

**TABLE 2:** Inflammatory signaling pathways involving primary cilia.

Study	Cell and tissue	Intervention	Ion channel	Results
Wann AK et al. (2014) [58]	Primary murine articular chondrocytes	IFT88 conditional knockout	NF-kB	Primary cilia influence the NF-κB signaling pathway by regulating IKK activity
Meng et al. (2023) [59]	Primary bovine articular chondrocytes	YAP	NF-kB	Activation of YAP inhibits the IL-1β-induced NF-κB signaling pathway and the release of downstream molecules, such as NO and PGE2, by regulating IKK activity
Hattori et al. (2021) [60]	Primary bovine articular chondrocytes	TRPV4 agonist (GSK1016790 A)	TRPV4 NF-kB	GSK101 increased AMPK phosphorylation and reduced IL-1β-induced NF-κB phosphorylation, reversing the increase in MMP-13 expression and the decrease in AGC and SOX9 expression
Fu et al. (2021) [61]	Primary bovine articular chondrocytes	TRPV4 agonist (GSK1016790 A), TRPV4 antagonist (GSK205)	TRPV4	GSK101 inhibited IL-1β-mediated NO release, while GSK205 promoted IL-1β-mediated release of NO and PGE2
O'Conor CJ et al. (2014) [62]	Primary porcine articular chondrocytes	TRPV4 agonist (GSK1016790 A), TRPV4 antagonist (GSK205)	TRPV4	GSK101 reduced the expression of NOS2 and ADAMTS5 genes, while enhancing the expression of COL2 and s-GAG
Takeda et al. (2021) [63]	ATDC5 mouse chondrogenic cell	TRPV4 agonist (GSK1016790A), TRPV4 antagonist (HC-067047)	TRPV4	GSK101 inhibited the compression load-induced mRNA levels of ADAMTS4 and IL-1R, whereas TRPV4 antagonists enhanced the mRNA levels of ADAMTS4 and IL-1R
Agarwal et al. (2021) [64]	Primary human articular chondrocytes	TRPV4 agonist (GSK101), TRPV4 antagonist (GSK205)	TRPV4	GSK101 increased the phosphorylation of GSK3β, promoting the expression of matrix metalloproteinases MMP3 and MMP13, as well as the inflammatory factors IL-6 and IL-8
Sun et al. (2022) [65]	Human synovium tissue SD rats RAW264.7 cells	TRPV4 agonist (GSK1016790A), TRPV4 antagonist (HC-067047)	TRPV4	The infiltration of M1 synovial macrophages and the expression of TRPV4 are significantly increased in OA synovium. The TRPV4 inhibitor HC067074 can alleviate the progression of OA in rats and significantly reduce the M1 polarization of synovial macrophages
Shen et al. (2023) [66]	Primary rat chondrocytes	TRPV4 antagonist	TRPV4	After TRPV4 inhibition, the expression of TRPV4, MMP-13, ADAMTS-5, and NOS2 mRNA in chondrocytes under inflammatory conditions significantly decreased, while the expression of COL2 and ACAN mRNA increased
Subramanian et al. (2017) [67]	Primary bovine articular chondrocytes	Chloral hydrat	MAPK	In deciliated chondrocytes, the expression of phosphorylated ERK1/2 is reduced
He et al. (2016) [68]	Primary human articular chondrocyte, C28/I2 chondrocytes, C57BL/6 mice	IFT88 siRNA	MAPK	Knockout of IFT88 reduced the mechanical load-induced expression of CITED2 and upregulated MMPs
Xiang et al. (2019) [69]	chondrocytic cell line ATDC5, primary rat chondrocytes	Icariin	MAPK	Icariin enhances ERK phosphorylation and increases the expression of COL2 and SOX9 by promoting primary cilia assembly and IFT88 expression, thereby maintaining the chondrocyte phenotype
Thompson et al. (2016) [70]	Primary bovine articular chondrocytes, Wistar rats	LiCL	Hedgehog	LiCl inhibits the Hedgehog signaling pathway, a process associated with the increase in primary cilia length
Ma et al. (2012) [71]	Primary human and bovine articular chondrocytes	IL-1β	Wnt/β-catenin NF-κB	The Wnt/β-catenin signaling pathway counteracts IL-1β-induced NF-κB-mediated MMPs expression through a negative feedback loop
Hdud et al. (2014) [72]	Primary equine articular chondrocytes	Osmotic pressure	TRPV4 MAPK	The expression of TRPV4 and BKCa channels is sensitive to changes in osmotic pressure, and these changes may involve the activation of ERK and p38

### NF-κB signaling pathway

IL-1β activates a wide range of intracellular inflammatory signaling, largely regulated through either NF-κB signaling, or mitogen activated kinase (MAP) activates or combinations of both. In OA models, damage or dysfunction of primary cilia is often associated with inhibition of the NF-κB pathway [73]. Numerous studies have shown that changes in ciliary proteins, such as IFT88, lead to alterations in the cytoskeletal structure. Since NF-κB relies on an active, dynein-dependent cytoskeletal transport system, it is particularly susceptible to the activation of cytokine-induced pathways [58]. Research by Wann et al. [58, 73]. Revealed how primary cilia influence inflammatory signaling via the NF-κB pathway. The disruption of primary cilia is associated with specific molecular events in the IL-1β-induced NF-κB pathway, leading to the inhibition of COX2 (cyclooxygenase-2) and iNOS (inducible nitric oxide synthase) protein expression [73]. Moreover, although IKK (IκB kinase) activation remains unaffected in cilia-deficient cells, the reduction in IκB phosphorylation delays and decreases IκB degradation, thus affecting the nuclear translocation and binding of NF-κB p65 [58]. Rose BJ et al. [57] also identified the potential key role of heat shock protein 27 (hsp27), which is linked to IKK activity and may be crucial for the regulation of the NF-κB pathway in primary cilia. Meng et al. [59] further demonstrated that YAP signaling regulates NF-κB pathway activation by inhibiting primary cilium expression, thereby blocking IL-1β-induced cartilage matrix degradation and exerting anti-inflammatory effects. However, dysfunction or loss of primary cilia can impair the ability of YAP to regulate the NF-κB pathway.

In conclusion, the disruption of the ciliary protein IFT88 and the loss of primary cilia alter the NF-κB-dependent response to inflammatory signals [58]. Therefore, ciliary proteins in the peripheral cytoplasm may represent a novel component in inflammatory signaling encoding, making them an exciting target for exploring methods to restore physiological inflammation signals in disease processes.

### TRPV4 signaling pathway

Mechanical stimuli have anti-inflammatory effects in many tissues [23]. TRPV4, located on the ciliary membrane [74], is one of the primary ion channels that senses mechanical stimuli through primary cilia, detecting mechanical forces in the joint, such as compression, stretching, or fluid shear stress [21]. While TRPV4 channels are widely distributed on the cell membrane of chondrocytes and not limited to primary cilia, the integrity of primary ciliary structure and function appears to play a significant role in TRPV4-mediated calcium responses [75]. Hattori et al. [60] demonstrated that activation of TRPV4 protects joint cartilage through the CaMKK/AMPK/NF-κB signaling pathway. Activation of TRPV4 inhibits the increase in MMP-13 expression induced by IL-1β and the decrease in AGC and SOX9 expression, alleviating cartilage degeneration and inflammation. Similarly, Fu et al. [61] showed that mechanical stimulation activates TRPV4 channels, enhancing HDAC6 expression and ciliary length, thereby mitigating IL-1β-induced NO release, inflammatory responses and cartilage degradation, which positively influences cartilage health. Furthermore, activation of TRPV4 channels in chondrocytes under dynamic compression load promotes anabolic processes and suppresses inflammation [62]. TRPV4 agonists suppress the expression of ADAMTS4 and IL-1 receptor (IL-1R) induced by compressive loading, while TRPV4 antagonists exhibit the opposite effect [63]. These findings suggest that activation of the TRPV4 pathway holds therapeutic potential for inhibiting pro-inflammatory signaling and preventing cartilage degradation in OA.

Other studies, however, have shown that activation of the TRPV4 channel exacerbates the progression of osteoarthritis. Activation of the TRPV4 ion channel increases intracellular calcium levels, which regulate the phosphorylation of GSK3β and inhibit its activity, impairing the ability of chondrocytes to sense the viscoelasticity of the extracellular matrix (ECM) [64]. Furthermore, inactivated GSK3β has been shown to upregulate several transcription factors, including β-catenin, c-Jun, and NF-κB. This upregulation promotes inflammatory responses and cartilage degradation [64, 76]. Sun et al. discovered that M1 synovial macrophage infiltration and TRPV4 expression were significantly increased in OA synovium. Conversely, TRPV4 inhibitors markedly reduced M1 polarization of synovial macrophages, thereby alleviating OA progression, potentially through the ROS/NLRP3 pathway [65]. Additionally, TRPV4 inhibitors downregulate the expression of genes associated with chondrocyte degeneration. In chondrocytes under inflammatory conditions, treatment with TRPV4 inhibitors leads to increased expression of ACAN and COL2, while reducing the expression of MMP13, ADAMTS5, and NOS2 [66].

The role of TRPV4 may be closely related to the timing of its activation, the physiological state of chondrocytes, and the stage of disease progression. Further studies are needed to explore the mechanistic details of TRPV4 and develop precise strategies to regulate its activity in OA treatment to optimize therapeutic outcomes. Overall, these findings highlight the significant role of primary cilia in regulating chondrocyte inflammatory responses via TRPV4 channels.

### MAPK signaling pathway

Deciliated chondrocytes exhibit lower basal expression levels of phosphorylated ERK1/2, suggesting that primary cilia may participate in signal transduction through the MAPK/ERK signaling pathway [67]. Studies have shown that under moderate mechanical stimulation, primary cilia, acting as cellular mechanosensors, activate the expression of CITED2 via the ERK1/2 phosphorylation. Such activation downregulates the transcription and expression of matrix-degrading enzymes MMP-1 and MMP-13,thereby exerting an anti-catabolic effect [68]. Additionally, drugs like Icariin [69] and bFGF [77] activate the ERK pathway by enhancing IFT88 expression, inducing the expression of SOX9 and COL2 genes, and promoting cartilage matrix secretion, thereby stimulating chondrocyte proliferation and differentiation while inhibiting inflammatory factors such as MMP3 and MMP9. Xiang et al. [78] showed that after sensing mechanical signals, primary cilia can activate the ERK pathway, further inhibiting the downstream mTOR signaling axis, which not only regulates chondrocyte autophagic activity but also alleviates cartilage degeneration by modulating the expression of inflammatory mediators. The platelet-derived growth factor receptor (PDGFRα), a G-protein-coupled receptor located on primary cilia, activates through binding with PDGF ligands, inducing cellular responses via the downstream MEK/ERK signaling cascade [79].

### Hedgehog signaling pathway

The Hedgehog signaling pathway consists of ligands like Sonic Hedgehog (Shh), Indian Hedgehog (Ihh), and Desert Hedgehog (Dhh), and receptors such as Patched (Ptc) and Smoothened (Smo). In the absence of Hedgehog ligands, Ptc inhibits Smo, but upon ligand binding to Ptc, this inhibition is relieved, activating downstream Gli transcription factors and influencing target gene expression [70, 80]. Hedgehog signaling plays a vital role in regulating cell proliferation, differentiation, and tissue morphogenesis [80, 81].

Numerous studies have demonstrated that Hedgehog signaling in chondrocytes requires primary cilia [82], and deletion of IFT88 disrupts primary cilia-mediated Hedgehog signaling [83]. IFT88 maintains the threshold level of Hh signaling under physiological mechanical loading, thereby regulating chondrocyte mineralization [83]. Thompson et al. [84] showed that mechanical loading activates primary cilia-mediated Hedgehog signaling in chondrocytes and promotes ADAMTS-5 expression, emphasizing the close link between primary ciliary length and Hedgehog signaling. This further underscores the importance of primary ciliary structure in Hedgehog signaling transduction and cartilage health. Hedgehog-related genes play key roles in regulating extracellular matrix and inflammatory responses and are involved in the pathogenesis of OA. Yang et al. [85] showed that upregulation of Ihh expression promotes chondrocyte hypertrophy and increases the expression of hypertrophic markers such as collagen X and MMP-13, further promoting cartilage matrix degradation and OA progression. In articular cartilage, IL-1β-induced ciliary elongation may also affect cilia function including mechanotransduction and Hedgehog signaling [86]. In their investigation of the potential of LiCl treatment for OA, Thompson et al. [80] found that LiCl significantly inhibited the Hedgehog signaling pathway, a process directly related to changes in ciliary length. Previous reports have shown that LiCl can suppress IL-1β-induced NF-κB signaling [87]. These studies suggest that LiCl may modulate ciliary length to influence the Hedgehog pathway, thereby playing a role in the regulation of inflammatory signaling.

In summary, the activation and transmission of Hedgehog signaling depend on the structure and function of primary cilia, and changes in primary cilia may be influenced by inflammatory factors like IL-1β. This interaction may play a role in various pathological processes, including the onset and progression of OA.

### Cross-regulation of signaling pathways

Primary cilia also engage in cross-regulation with various signaling pathways. According to a review by Pala et al. [88], primary cilia are involved in regulating several signaling pathways, including Hedgehog, Wnt, PDGFR, Notch, and TGF-β. The interactions between these pathways may jointly influence the regulation of inflammation. For example, the interaction between Wnt/β-catenin and NF-κB signaling has been well-documented [89]. Wnt/β-catenin not only reduces the basal expression levels of MMP1, MMP3, and MMP13 but also inhibits NF-κB-induced upregulation of MMPs in human chondrocytes [71, 90]. This indicates that the Wnt/β-catenin pathway can antagonize NF-κB signaling and exert a protective effect. Similarly, activation of TRPV4 can inhibit IL-1β-induced cartilage degeneration and inflammatory responses by activating the CaMKK/AMPK pathway and suppressing NF-κB activation. This process plays a key role in cartilage degradation and the progression of osteoarthritis [60]. Additionally, when chondrocytes are exposed to hypotonic conditions, the expression of TRPV4 channel proteins rapidly increases, triggering a MAPK cascade that leads to ERK1/2 phosphorylation, and in turn, ERK1/2 phosphorylation regulates the endogenous expression of TRPV4 channels. These two processes may interact with each other in a feedback loop [72]. Furthermore, the natural isoflavone glycoside ononin alleviates IL-1β-induced reductions in chondrocyte viability by concurrently downregulating MAPK and NF-κB signaling pathways. Ononin also mitigates the overexpression of inflammatory factors TNF-α and IL-6 and reverses extracellular matrix degradation by inhibiting MMP-13 expression and promoting COL2 expression, thereby improving chondrocyte inflammation [38].

These findings collectively demonstrate the complexity and diversity of the inflammatory response in osteoarthritis. The cross-regulation of multiple signaling pathways involving primary cilia profoundly influences the inflammatory processes associated with osteoarthritis.

## Mechanisms of inflammatory damage to primary ciliary function

Primary cilia are involved in modulating inflammatory responses in chondrocytes. When chondrocytes are stimulated by inflammatory cytokines like IL-1β, primary cilia may regulate their function by adjusting ciliary length, a process potentially controlled by IFT protein activity [74]. Primary cilia and IFT (intraflagellar transport) are key in inflammatory signal transduction [86]. IL-1 is one of the most important inflammatory mediators in the formation and progression of OA [23]. Research by Wann et al. [86] showed that exposure to IL-1 for 3 hours increased primary ciliary length by 50% in chondrocytes. This elongation occurs via a protein kinase A (PKA)-dependent mechanism. Furthermore, IL-1-treated chondrocytes exhibited increased release of typical inflammatory mediators such as NO and PGE2. However, in cells with IFT88 mutations leading to ciliary structural loss, the IL-1-induced inflammatory response was significantly diminished, and the progression of OA was correspondingly reduced. These findings suggest that primary cilia are involved in regulating intracellular inflammatory responses.

Histone deacetylase 6 (HDAC6) is a major driver of ciliary disassembly. HDAC6 can induce ciliary disassembly through the deacetylation of α-tubulin and cortactin [91]. Fu [92] and Zhang’s studies [93] indicated that inhibiting HDAC6 promotes microtubule assembly and active elongation, improving the IL-1β-induced inflammatory response. They proposed that the inflammatory environment promotes passive elongation of primary cilia to enhance their mechanotransduction effects, enabling chondrocytes to adapt optimally to inflammation. In contrast, when HDAC6 is inhibited, primary cilia actively elongate to improve mechanotransduction and adjust to their most favorable state.

In summary, primary cilia play a significant role in mediating inflammatory responses through morphological and functional changes. Interestingly, the elongation of primary cilia in response to IL-1 requires the accumulation of hypoxia-inducible factor-2α (HIF-2α) in the cilia [94]. Research by Yang et al. [95] showed that upregulation of HIF-2α promotes OA progression by mediating primary ciliary loss. Another study by Fu et al. [96] also indicated IL-1β-induced elongation of primary cilia in chondrocytes. However, other studies have reported a decrease in the incidence of primary cilia in chondrocytes following IL-1β treatment [75].

## Impact of primary cilia on synovitis

Primary cilia serve as sensors to detect changes in the osmotic pressure or chemical composition of synovial fluid and act as signaling transducers to regulate intracellular signaling pathways in chondrocytes [97]. In osteoarthritis, synovial inflammation is a key factor in joint destruction. Fibroblast-like synovial cells (FLS), the main cellular component of the synovium, may promote OA progression by producing pro-inflammatory mediators, such as inflammatory cytokines, nitric oxide (NO), and prostaglandin E2 (PGE2) [98]. FLS respond to the secretion of inflammatory factors by secreting cytokines into the synovial fluid, making them essential components in maintaining the articular cartilage environment [99]. Research by Yuan et al. [100] showed that during inflammatory arthritis, both the length and incidence of cilia in FLS were increased, a change that could enhance the transduction of inflammatory signals to regulate cell function, thereby triggering joint swelling and cartilage damage. Similarly, Estell et al. [99] found that IL-1α significantly increased the average length and incidence of primary cilia in FLS under OA conditions. FLS can sense and respond to mechanical and chemical stimuli in the joint, and changes in their function are associated with OA progression.

## Conclusions and perspectives

Inflammation is an integral factor in the pathogenesis of osteoarthritis. Various molecules released by chondrocytes, synovial cells, and immune cells, including cytokines, chemokines, and MMPs, are involved in maintaining cartilage homeostasis [25]. At the same time, the expression of these molecules is regulated by signaling pathways such as NFκB, MAPK, PI3K/AKT, prostaglandins, and nitric oxide [25]. Primary cilia, as sensory organelles, have the ability to detect and regulate physical and chemical signals, making them important mechanoreceptors [74, 101]. There is a close relationship between inflammatory responses, mechanical loading, and cartilage homeostasis [102]. Therefore, targeting the receptors of inflammatory mediators and the mechanotransduction mechanisms of chondrocytes primary cilia may be an effective strategy for directly controlling chondrocyte responses to pathological loads or disease progression [102].

Despite existing research revealing the potential role of primary cilia in inflammatory responses in osteoarthritis, many scientific questions remain unresolved. For example, it is still unclear how primary cilia mediate bidirectional regulation of chondrocyte degradation and regeneration, how primary cilia act as a bridge between different signaling pathways, and how to modulate the function of primary cilia to suppress inflammation and repair cartilage. In conclusion, primary cilia play an important role in the inflammatory response of osteoarthritis and may represent a novel therapeutic target for treating osteoarthritis in the future.
